# Para-Aortic Lymph Node Staging and Oncologic Outcomes in Locally Advanced Cervical Cancer: A Narrative Review

**DOI:** 10.3390/cancers18132058

**Published:** 2026-06-25

**Authors:** Juan Sebastián Obando-Rodríguez, Santiago Vieira-Serna, Jonathan Peralta, Juliana Rodríguez, Erick Estrada, Luisa López-Saldarriaga, Gabriel Levin, Rene Pareja

**Affiliations:** 1 Department of Gynecologic Oncology, Instituto Nacional de Cancerología, Bogotá D.C. 111511, Colombia; 2 Faculty of Medicine, Universidad Militar Nueva Granada, Bogotá D.C. 110111, Colombia; 3 Grupo de Investigación Salud de la Mujer Sanitas, Department of Gynecologic Oncology, Clínica Colsanitas S.A., Clínica Universitaria Colombia, Bogotá D.C. 111321, Colombia; 4 Division of Gynecologic Oncology, Department of Obstetrics and Gynecology, Fundación Santa Fe de Bogotá, Bogotá D.C. 110111, Colombia; 5 Department of Obstetrics and Gynecology, Universidad Nacional de Colombia, Bogotá D.C. 111321, Colombia; 6 Hospital General San Juan de Dios Ciudad de Guatemala, Ciudad de Guatemala 01001, Guatemala; 7 Intergastro S.A., Medellín 050021, Colombia; 8 Division of Gynecologic Oncology, McGill University Health Centre, McGill University, Montreal, QC H4A 3J1, Canada

**Keywords:** uterine cervical neoplasms, lymph node excision, neoplasm staging, locally advanced cervical cancer, diagnostic imaging, chemoradiotherapy

## Abstract

**Background**: Para-aortic lymph node involvement is present in approximately 17–24% of women with locally advanced cervical cancer (LACC) and is one of the strongest adverse prognostic factors in this population. Current international guidelines recommend two alternative staging techniques: the International Federation of Gynecology and Obstetrics (FIGO) and European Society of Gynecologic Oncology (ESGO) endorse imaging-based staging as the primary method to define radiation fields, whereas the National Comprehensive Cancer Network (NCCN) lists pre-treatment minimally invasive para-aortic lymphadenectomy as a Category 2B recommendation. **Objective**: We aimed to review and critically appraise the available evidence on the oncologic impact (progression-free and overall survival) of pre-treatment surgical para-aortic staging compared with clinical imaging-based staging in women with LACC. **Methods**: We searched MEDLINE (Ovid), Embase, the Cochrane Central Register of Controlled Trials (CENTRAL), ClinicalTrials.gov, and Scopus from inception to January 2026, complemented by manually searching the reference lists for relevant articles and prior reviews. The review focused on comparative studies of women with LACC of squamous, adenocarcinoma, or adenosquamous histology—operationally defined as FIGO 2009 stages IB2–IVA with pelvic nodal involvement or FIGO 2018 stages IB3–IVA who received definitive-intent radiotherapy with or without concurrent chemotherapy and brachytherapy, and for whom comparative survival outcomes between a surgical-staging arm and an imaging-staging arm were reported. For this manuscript, a narrative review style was planned and reported in line with SANRA (Scale for the Assessment of Narrative Review Articles) quality criteria. **Results**: Twelve studies were included: two randomized controlled trials and ten observational studies (nine retrospective cohorts and one population-based analysis). Surgical staging consistently increased detection of occult para-aortic disease and led to more frequent use of extended-field radiotherapy (18–44%), but it did not yield a reproducible advantage in terms of progression-free or overall survival over imaging-guided chemoradiation. **Conclusions**: In LACC, pre-treatment surgical para-aortic staging improves anatomic and prognostic information but has not shown a consistent survival advantage over imaging-based staging combined with contemporary chemoradiation. Current comparative evidence does not support routine surgical staging, and its use still warrants further prospective evaluation in large clinical trials. Until results from ongoing phase III trials are available, surgical staging should be considered an individualized option in highly selected cases within multidisciplinary decision-making at experienced clinical centers.

## Simple Summary

Locally advanced cervical cancer (LACC) remains a major cause of cancer-related morbidity, particularly in low- and middle-income countries. Approximately 17–24% of women with LACC harbor metastatic disease in the para-aortic lymph nodes at diagnosis, a finding that constitutes one of the strongest adverse prognostic factors and directly influences the cranial extent of the radiation field. Two staging strategies are currently employed in clinical practice: para-aortic lymphadenectomy with histopathological assessment of the retrieved nodes, and imaging-based staging. For decades, clinicians have debated which staging strategy yields superior oncological outcomes, and current international guidelines provide recommendations supporting both approaches. In this narrative review, we summarize and critically appraise the scientific evidence available to date, comparing surgical with imaging-based staging in women with LACC with respect to oncological outcomes. Our goal is to help clinicians, guideline developers, and trial designers understand what the current evidence supports and to identify key questions that ongoing trials must still answer.

## 1. Introduction

To date, cervical cancer remains a challenging global health problem, ranking as the fourth most common malignancy among women, with approximately 662,000 new cases and 349,000 deaths reported worldwide in 2022, disproportionately burdening low- and middle-income countries [1]. Incidence and mortality are inversely related to a country’s Human Development Index: low-HDI countries face nearly three times the incidence and six times the mortality of very-high-HDI countries [2,3]. This regional disparity is particularly relevant to the present review, because pre-treatment surgical para-aortic staging is largely studied in, and proposed for, high-resource health systems, while the patients who most often present with locally advanced disease live in countries where access to PET-CT, image-guided brachytherapy, and minimally invasive surgical expertise is constrained [1]. In locally advanced cervical cancer (LACC; FIGO 2009 stages IB2–IVA or FIGO 2018 stages IB3–IVA), para-aortic lymph node (PALN) involvement is present in approximately 17–24% of cases [4] and constitutes the single most important prognostic factor in this population, with a five-year overall survival of approximately 37% [5,6]. Conceptually, para-aortic involvement has historically been managed as a loco-regional event guiding the cranial extent of the radiation field; however, some evidence suggests that it may behave more as a marker of systemic disease biology, with a major influence on patterns of distant recurrence [7]. The current standard primary therapy for LACC is cisplatin-based concurrent chemoradiotherapy (CCRT) combined with image-guided adaptive brachytherapy [8]. Target therapies such as immune checkpoint inhibitors (ICI) are now also part of the treatment arsenal for patients with LACC, especially for those cases with high-risk features such as stages III-IVA [9]. Documented para-aortic involvement, whether radiologic or pathologic, requires extended-field external-beam radiotherapy (EBRT) up to the level of the renal vessels [8,10]. Current major international guidelines diverge slightly in staging strategy recommendations; FIGO and ESGO endorse imaging-based staging—preferably 18F-Fluorodeoxyglucose Positron Emission Tomography–Computed Tomography (18F-FDG PET-CT), with Magnetic Resonance Imaging (MRI) or Computed Tomography (CT) as alternatives when PET-CT is unavailable—as the primary method to define the cranial extent of the radiation field [8,10]. In contrast, NCCN lists pre-treatment laparoscopic or extraperitoneal para-aortic lymphadenectomy as a Category 2B option—that is, included as an accepted alternative on the basis of lower-level evidence and non-uniform expert consensus [11].

Identifying para-aortic nodal metastasis is key due to the rationale behind determining the appropriate upper extent of the radiation field on the abdomen. The most recent international guidelines therefore recommend elective para-aortic extended-field radiation in patients meeting EMBRACE-II nodal-burden criteria [10,11]. According to the EMBRACE-I protocol, extension of the external beam radiation therapy (EBRT) field to include coverage of the para-aortic region is indicated in two main scenarios: first, when there is radiologically or histologically confirmed para-aortic lymph node involvement, and second, in patients considered at high risk of having unnoticed nodal involvement. Patients considered at high-risk were determined at the discretion of the treating physician, and this decision was based on clinical imaging findings, but unfortunately, the protocol did not specify rigid criteria for “high risk” [12]. The aforementioned was one of the main reasons for the development of the EMBRACE-II protocol, an international prospective study protocol that represents the current state-of-the-art treatment approach for locally advanced cervical cancer and has been adopted as standard practice at multiple institutions worldwide [13]. This protocol combines external beam radiation therapy (EBRT), concurrent cisplatin chemotherapy, and MRI-guided adaptive brachytherapy with highly standardized technical specifications. The definition of “high-risk” disease in this protocol is based on either the presence of at least one suspicious node at pelvic nodal level 2 (common iliac or presacral nodes) or three or more suspicious nodes at pelvic nodal level 1 (hypogastric, obturator or external iliac vessels), as these findings are major predictors of para-aortic and systemic failure [14]. Based on real-world data from patients treated with the EMBRACE-II protocol, the 5-year para-aortic control rate was 93%, and the 5-year systemic (distant) control rate was 82% [15].

The detection of paraaortic nodal involvement through surgical staging has important prognostic implications, but to date, it has yet to be proven in prospective evaluation to improve oncological outcomes. Additionally, surgical para-aortic lymph node staging has been shown to delay treatment initiation in some retrospective studies, but the delay appears to be modest (approximately 10–18 days), and most importantly, it does not seem to adversely affect survival outcomes in study populations [16]. On the other hand, clinical staging with modern imaging offers the advantage of being non-invasive and less likely to delay initiation of chemoradiation. However, even 18 Fluorodeoxyglucose (18F-FDG)–PET/CT underestimates para-aortic lymph node metastasis, failing to detect micro metastases below 5 mm and missing para-aortic disease in roughly 10–15% of overall patients, with false-negative rates rising to 20–30% among those with pelvic nodal uptake, which may leave a substantial proportion of high-risk patients undertreated [17,18,19]. PET-CT is also an expensive and unevenly distributed imaging modality across the regions in which LACC is most prevalent [20]. By summarizing the available comparative evidence, highlighting persistent gaps, and describing ongoing trials such as PAROLA [21] and CQGOG0103 [22], this review aims to inform contemporary practice, guideline updates, and the design of future trials.

## 2. Materials and Methods

This narrative review aims to summarize and critically appraise the available comparative evidence on the oncologic impact (progression-free and overall survival) of pre-treatment surgical para-aortic staging versus imaging-based staging in women with LACC. We identified relevant comparative studies—both randomized and observational—through a literature search across MEDLINE, Embase, CENTRAL, ClinicalTrials.gov and Scopus from inception to January 2026, complemented by manually searching the reference lists for relevant articles and prior reviews. We focused on studies of women with LACC (FIGO 2009 IB2–IVA or FIGO 2018 IB3–IVA) treated with definitive radiotherapy with or without concurrent chemotherapy and brachytherapy, in which survival outcomes were reported separately for a surgical-staging and an imaging-staging arm. We identified 473 records (PubMed 156; Scopus 144; Embase 118; Cochrane 43; ClinicalTrials.gov 12). After removal of duplicates, 234 records were screened based on title and abstract, and 110 reports were assessed in full text for eligibility. After final assessment, twelve comparative studies (two randomized controlled trials and ten observational studies) ultimately met the criteria for inclusion. Screening and full-text assessment were performed independently by two authors (J.S.O.-R. and L.L.-S.), with disagreements resolved by a third reviewer (R.P.). For the purposes of this synthesis, we did not include single-arm series, studies limited to diagnostic accuracy, feasibility, or upstaging rate without survival outcomes, or studies in which the para-aortic dissection did not extend at least to the inferior mesenteric artery.

Studies are presented first by design and then chronologically, with a qualitative appraisal of each study’s applicability to contemporary practice. This review was planned and reported in line with the SANRA (Scale for the Assessment of Narrative Review Articles) quality criteria, ensuring a clear statement of importance and aims, transparent description of the literature search, appropriate referencing, explicit presentation of evidence levels, and reporting of relevant endpoint data [23].

Use of Generative Artificial Intelligence: During the preparation of this manuscript, the authors used Claude (Claude Sonnet 4.6, Anthropic, San Francisco, CA, USA) for the purposes of language editing (grammar, spelling) and for assistance with the formatting, layout, and visual organization of the summary tables. All scientific content—including the literature search, study selection, data extraction, critical appraisal of each included study, interpretation of findings, and conclusions—was developed by the authors. The authors have reviewed and edited the output and take full responsibility for the content of this publication.

## 3. Results

We discuss twelve comparative studies of pre-chemoradiation surgical versus imaging-based para-aortic staging in LACC: two randomized controlled trials and ten observational studies (nine retrospective cohorts and one population-based analysis of the U.S. National Cancer Data Base [NCDB]). To prioritize the level of evidence, studies are presented first by design—randomized controlled trials, followed by observational studies—and chronologically within each group. This structure also allows the reader to follow the temporal evolution of staging and treatment standards. In general, surgical staging consistently increased the detection of occult para-aortic disease (upstaging in 8–48% of patients) and led to more frequent use of extended-field radiotherapy (18–44%), but the survival comparisons varied substantially across studies and were heavily influenced by patient selection, by the contemporaneity of radiation and systemic therapy, and by the surgical approach used (Table 1).

**Table 1:** Main characteristics of studies comparing pre-treatment surgical and imaging-based para-aortic lymph-node staging in locally advanced cervical cancer.

Study	Design/Period	n	FIGO/Histology	Staging Arms	RT/Surgical Details	Detection and Treatment Changes	Survival Outcomes
***Randomized** **Controlled Trials***							
**Lai et al., 2003** [24]	RCT 1993–2000	61	IIB–IVA Sq, AdCa, AdSq	Surg (n = 32): EXP laparotomy or LAP Clin (n = 29): CT/lymphangiogram	Cisplatin/5-FU CRT 4-field RT; variable BT	PALN+ 25% (Surg) Trial closed early at interim analysis	PFS HR 3.13 (1.42–6.89), *p* = 0.005 OS *p* = 0.024; both favoring clinical arm
**Marnitz et al., 2020** **(UTERUS-11)** [6]	Phase III RCT 2009–2013 (90-mo follow-up)	240 (255 enrolled)	IIB–IVA Sq, AdCa, AdSq	Surg (n = 121): transperitoneal LAP to renal vessels Clin (n = 119): imaging	50.4 Gy EBRT (65% IMRT) HDR-BT 94.5% Weekly cisplatin 97.5%	Upstaging: 33% (Surg) vs. 8% (Clin) EFRT: 23% vs. 12% (*p* = 0.02) Intraop complications: 1.6%	PFS HR 0.73 (0.49–1.08), *p* = 0.084 (NS) OS: NS Post hoc CSS HR 0.61 (0.40–0.92), *p* = 0.018
***Retrospective Cohorts***							
**Gold et al., 2008** [25]	Post hoc pooled analysis 3 GOG RCTs (GOG-85/120/165)	685	IIB–IVA Sq, AdCa	Surg (n = 555): mandatory open EXP PALND Clin (n = 130): CT/MRI/lymphangiography	4-field RT; BT omitted >10% Obsolete 5-FU/hydroxyurea regimens	Fewer PA recurrences (Surg) Comparison not pre-specified	4-yr PFS: 48.9% vs. 36.3% 4-yr OS: 54.3% vs. 40.0% Multivariable HR favoring surgical arm
**Chen et al., 2012** [26]	Single-center retrospective 1993–2001	56	IIB–IVA (elderly ≥ 65 y) Sq only	Surg (n = 19): transperitoneal LAP PALND Clin (n = 37): imaging	RT or CCRT (only 23% CCRT) Historical protocols	Occult PALN+: 15.8% (3/19) Major bowel complications: 36.8%	Median FFS: 44 vs. 23 mo, *p* = 0.276 Median OS: 51 vs. 24 mo, *p* = 0.372
**Vandeperre et al., 2015** [27]	Single-center retrospective 1994–2013	336	IB2–IVA 83% Sq	Surg (n = 204): retroperitoneal endoscopic PALND (all imaging-negative PA) Clin (n = 132): imaging only	CCRT, NACT, or radical hysterectomy 20-year accrual; evolving protocols	PET-CT false-negative rate: 11.1% CT false-negative rate: 18.9%	2-yr OS: 40% (sPALN+) vs. 83% (sPALN−) vs. 58% (iPALN+) vs. 69% (iPALN−), *p* < 0.001
**Chargari et al., 2016** [28]	Single-center retrospective 2004–2012	186	IB2–IVA Mixed histology	PAL (n = 119): PET-CT-negative + retroperitoneal PALND No-PAL (n = 50): PET-CT-negative, imaging only	Cisplatin/carboplatin CCRT MRI-based IGABT EFRT only if PALN+	Occult PALN+: 7.6% overall; 15.8% if pelvic PET uptake Tailored field extension in PALN+ cases	3-yr DFS: 80% vs. 58%, *p* < 0.05 Distant FFS: 87% vs. 67% Multivariable DFS favoring PAL arm
**Pomel et al., 2017** [29]	Bi-center retrospective 2001–2013	187	IB2–IVA Mixed histology	Center 1 (n = 98): PET-CT only Centre 2 (n = 89): routine LAP PALND	Cisplatin CCRT + BT BT use: 94.9% (Clin) vs. 76.5% (Surg)	Staging confounded with centre Baseline imbalances (age, PS, BT use)	5-yr DFS: 70.5% (Clin) vs. 49.2% (Surg), *p* = 0.009 5-yr OS: 85.1% vs. 63.8%, *p* = 0.006
**Dabi et al., 2018** [30] **(FRANCOGYN)**	Multicentre retrospective 1996–2016	647	IB2 or higher Mixed histology	Surg (n = 377): LAP PALND (imaging-negative PA) Clin (n = 270): imaging only (CT 42.7%; PET-CT 57.3%)	Pelvic CCRT; BT 53% PA RT boosts 23.6%	Occult PALN+: 12.5% (47/377) Median follow-up: 38.1 mo	Multivariable analysis: no significant survival difference between arms
**González-Benítez et al., 2019** [31]	Single-center retrospective 2005–2012	74	IIA2–IVA Mixed histology	Surg (n = 43): LAP PALND Clin (n = 31): CT, MRI, or PET-CT	CCRT ± EFRT Mean follow-up: 24.2 vs. 19.1 mo	PALN+: 48.8% (Surg) vs. 33.3% (Clin) Severe complications: 9.3% (Surg)	Median PFS: 14.4 vs. 17.4 mo, *p* = 0.456 OS: *p* = 0.676—no significant difference
**Yang et al., 2020** [32]	Retrospective PSM Mayo Clinic sites	105 (35 Surg/70 Img)	IB2–IIIB Mixed histology	Surg (n = 35): MIS or open pelvic ± PALND Clin (n = 70): CT, MRI, or PET-CT	CCRT + BT Consolidation chemo more frequent in Surg arm	Imaging sensitivity for PALN: 62.5% Staging modified treatment in 31.4% (Surg)	5-yr PFS: 62.6% vs. 72.4%, *p* = 0.77 5-yr OS: 70.2% vs. 70.5%, *p* = 0.96 PALN+ HR for OS: 2.9 (1.1–7.9)
**Jiang et al., 2024** [33]	Single-center retrospective 2014–2018	171	IB2/IIA2–IVA pelvic LN+ Mixed histology	Surg (n = 58): LAP pelvic + PALND Clin (n = 113): CT-based imaging	CCRT RT started 10.2 d later in Surg arm	PALN+: 34.5% (Surg) Imaging over-called pelvic LN in 12.2% Similar grade 3–4 toxicity	5-yr PFS: 70.7% vs. 64.6%, HR 0.80 (0.45–1.41), *p* = 0.43 5-yr OS: 75.9% vs. 69.7%, HR 0.74 (0.40–1.37), *p* = 0.34
***Population-Based Analysis***							
**Nasioudis et al., 2022** [34]	Population-based (NCDB) 2010–2015	3540	IB2–IVA Mixed histology	Surg PALND (n = 333; 9.4%) Clin/radiologic (n = 3207)	Standard-of-care CCRT No central radiology or pathology review	PALN+: 27.3% (Surg) vs. 13.2% (Clin) Diagnosis-to-RT interval: 55 d (Surg) vs. 38 d (Clin)	4-yr OS: 63% vs. 62.9%, *p* = 0.80 Multivariable OS HR 1.07 (0.88–1.31)

**Abbreviations:** BT, brachytherapy; CCRT, concurrent chemoradiotherapy; Clin, imaging-based/clinical staging arm; CSS, cancer-specific survival; DFS, disease-free survival; EBRT, external-beam radiotherapy; EFRT, extended-field radiotherapy; EXP, extraperitoneal; FFS, failure-free survival; FIGO, International Federation of Gynecology and Obstetrics; HDR, high-dose rate; HR, hazard ratio; IGABT, image-guided adaptive brachytherapy; Img, imaging arm; IMRT, intensity-modulated radiotherapy; iPALN, imaging para-aortic lymph node; LAP, laparoscopic; MIS, minimally invasive surgery; NACT, neoadjuvant chemotherapy; NCDB, National Cancer Data Base; NS, not significant; OS, overall survival; PA, para-aortic; PALN, para-aortic lymph node; PALND, para-aortic lymph node dissection; PFS, progression-free survival; PS, performance status; PSM, propensity score-matched; RCT, randomized controlled trial; RT, radiotherapy; Sq, squamous cell carcinoma; AdCa, adenocarcinoma; AdSq, adenosquamous carcinoma; sPALN, surgical para-aortic lymph node; Surg, surgical staging arm.

### 3.1. Randomized Controlled Trials

Lai et al. 2003 [24]. Lai and colleagues reported the first randomized controlled trial comparing pre-treatment surgical lymph-node staging with clinical (imaging-based) staging in LACC. The aim of the study was to determine whether surgical staging improved oncologic outcomes over imaging-based staging in women with FIGO IIB–IVA cervical cancer. After enrolling 61 patients (29 to clinical staging, 32 to surgical staging), patients in the surgical arm were further randomized to laparoscopy or to extraperitoneal open laparotomy. The trial was terminated early after an interim analysis showed significantly worse progression-free survival in the surgical arm (HR 3.13, 95% CI 1.42–6.89, *p* = 0.005). Although the hazard ratio for overall survival was initially non-significant at interim analysis (HR 1.76, 95% CI 0.81–3.79, *p* = 0.15), longer follow-up eventually revealed a statistically significant overall survival difference in favor of clinical staging (*p* = 0.024). However, this result published in the original manuscript arose from post hoc analyses of an underpowered, inconclusive study, and should therefore be interpreted as exploratory rather than definitive. Recurrence or persistence occurred in 66% of surgically staged vs. 34% of clinically staged patients despite similar rates of grade 3–4 toxicity. Para-aortic metastases were identified in 25% of surgically staged patients, and all eight patients with documented para-aortic involvement died. Among the six who developed recurrent or persistent disease, reported survival ranged from 6 to 31 months, while the remaining two died early from treatment-related complications during or shortly after chemoradiation, without definite evidence of persistent tumor. Important caveats include now-obsolete chemotherapy (cisplatin/5-fluorouracil/hydroxyurea regimens) and radiotherapy (four-field pelvic radiotherapy without brachytherapy in a proportion of patients) and unbalanced baseline characteristics (more stage IIIB and adenocarcinoma, less concurrent chemotherapy in the surgical group). The authors concluded that surgical staging did not improve outcomes and may have caused harm under those treatment conditions. This trial represented an early sign that routine surgical staging without modern systemic and radiation therapy was unlikely to confer a survival benefit.

Marnitz et al., 2020—UTERUS-11 [6]. The UTERUS-11 trial is the only contemporary phase III randomized trial of pre-treatment surgical versus clinical staging in LACC. This trial enrolled 255 women (240 evaluable) with FIGO 2009 IIB–IVA squamous, adenocarcinoma, or adenosquamous cervical cancer, who were randomized 1:1 to pre-chemoradiation laparoscopic surgical staging (n = 121) or to clinical staging with imaging ± CT-guided biopsy of suspicious para-aortic nodes (n = 119). The primary endpoint was progression-free survival; secondary endpoints included overall survival, cancer-specific survival, and morbidity. Surgical staging, performed almost entirely transperitoneally up to the renal vessels, was safe (1.6% intraoperative and 7.3% postoperative complications; 0.8% conversion), recovered a median of 17 para-aortic and 19 pelvic nodes, upstaged 33% of patients vs. 8% upstaging by imaging, and led to extended-field radiotherapy in 23% vs. 12% of patients, without significantly delaying chemoradiation (median 13 days post-surgery). All patients received high-quality concurrent chemoradiation (median 50.4 Gy EBRT, 65% IMRT; HDR brachytherapy in 94.5%, median 28 Gy; weekly cisplatin in 97.5%). After a median follow-up of 90 months, 102 events occurred; there was no significant difference in the primary endpoint of progression-free survival (HR 0.73, 95% CI 0.49–1.08, *p* = 0.084; 5-year ≈ 67% surgical vs. 57% clinical) or in overall survival. An exploratory post hoc analysis of cancer-specific survival favored the surgical arm (HR 0.61, 95% CI 0.40–0.93, *p* = 0.020), and an exploratory subgroup analysis suggested a PFS benefit in stage IIB patients (HR 0.51, 95% CI 0.30–0.86, *p* = 0.011; interaction *p* = 0.031), although these analyses were not pre-specified and should be strictly regarded as hypothesis-generating. Main limitations of this clinical trial include its inability to reach the statistical power planned (70% power achieved vs. 80% planned) because of fewer events than expected and the absence of routine PET-CT (only 47 scans across the entire cohort). The authors concluded that surgical staging is feasible, safe and improves upstaging detection but does not deliver a clear survival advantage over imaging-guided chemoradiation.

### 3.2. Retrospective Cohorts and Population-Based Analyses

Gold et al., 2008 [25]. In 2008, Gold and colleagues performed a retrospective post hoc analysis of three phase III GOG trials: GOG 85, GOG 120 and GOG 165 [35,36,37], all enrolling women with clinically staged FIGO IIB–IVA cervical cancer and negative or presumed-negative para-aortic nodes, in which para-aortic status had been established either by mandatory extraperitoneal para-aortic lymphadenectomy (GOG 85 and 120 plus a subset of GOG 165, n = 555) or by radiologic imaging alone, predominantly CT, in GOG 165 (n = 130). The primary outcomes of this pooled analysis were progression-free and overall survival. Patients in the imaging (“radiographic”) cohort had, on average, better baseline characteristics (higher performance status, less advanced FIGO stage, smaller tumors), yet their adjusted 4-year outcomes were numerically worse: progression-free survival was approximately 49% versus 36% and overall survival 54% versus 40% in favor of the surgically staged group, and multivariable models associated radiographic staging with a higher risk of progression (HR 1.35, 95% CI 1.01–1.81) and death (HR 1.46, 95% CI 1.08–1.99). Para-aortic recurrences were also more frequent in the imaging group, consistent with occult PALN metastases being missed by CT-based staging in a proportion of patients. However, surgical versus radiographic staging was neither randomized nor pre-specified in either one of this studies, and the comparison amalgamated heterogeneous, now obsolete protocols (5-fluorouracil- and hydroxyurea-based chemoradiation, four-field pelvic RT with ≥10% omission of brachytherapy, open extraperitoneal laparotomy and no PET-CT), introducing substantial selection and temporal bias that limits the applicability of this apparent survival advantage to contemporary minimally invasive staging and IMRT-based chemoradiation practice.

Chen et al., 2012 [26]. Chen and colleagues [26] reported a single-center retrospective cohort study of 56 elderly patients (≥65 years) with FIGO IIB–IVA squamous cervical cancer treated between 1993 and 2001 (FIGO version not specified in the original publication) at the Mackay Memorial Hospital in Taiwan. The aim was to compare oncologic outcomes between transperitoneal laparoscopic para-aortic lymph-node dissection (n = 19) and clinical/radiologic staging (n = 37) prior to definitive radiotherapy or chemoradiotherapy. Surgical staging identified positive para-aortic nodes in 15.8% of patients (3 of 19). Patients in the surgical arm received more intensive treatment (concurrent chemoradiation 42.1% vs. 13.5%; extended-field radiotherapy 15.8% vs. 2.7%), yet no statistically significant survival differences were observed: median failure-free survival 44 vs. 23 months (*p* = 0.276), median overall survival 51 vs. 24 months (*p* = 0.372). The only statistically significant between-group difference was an excess of subsequent major bowel complications—fistula, obstruction, or radiation colitis requiring colectomy—in the surgical arm (36.8%, 7 of 19) compared with the control arm (10.8%, 4 of 37; *p* = 0.032), which the authors attributed to the transperitoneal procedure itself rather than to the radiotherapy modality, leading them to propose extraperitoneal lymphadenectomy as a safer alternative for elderly patients. This manuscript contains important methodological limitations—small sample size, transperitoneal access technique (now considered obsolete), selection bias (restriction to elderly population) and only 23% concurrent chemoradiation uptake, reflecting an era before CCRT became standard in 1999—so valid conclusions are impossible to obtain.

Vandeperre et al., 2015 [27]. Vandeperre and colleagues retrospectively analyzed 336 women with FIGO IB2–IVA cervical cancer treated between 1994 and 2013, all staged with imaging (PET-CT, PET plus CT, or CT alone), of whom 204 with negative para-aortic nodes underwent retroperitoneal laparoscopic para-aortic lymphadenectomy up to the inferior mesenteric artery. The aim of the study was to compare oncologic outcomes between groups defined by para-aortic status determined surgically versus radiologically. Patients were stratified into four groups according to para-aortic status by surgery and imaging: surgical para-aortic lymph node positive (sPAOLN^+^), surgical para-aortic lymph node negative (sPAOLN^−^), imaging para-aortic lymph node positive (iPAOLN^+^) and imaging para-aortic lymph node negative (iPAOLN^−^). Despite negative imaging, 8% of surgically staged patients harbored para-aortic metastases. Using histopathology of the surgical specimen as the reference standard, the patient-level false-negative rate of imaging—calculated among patients with truly negative imaging who successfully underwent surgical staging—was 3 of 64 for PET-CT (5%), 6 of 46 for PET plus separate CT (13%), and 4 of 68 for CT alone (6%); differences between modalities were not statistically significant. The overall patient-level false-negative rate, including the 10 patients with imaging classified as “suspicious but not overtly malignant,” was 16 of 204 (8%). Another relevant finding was that para-aortic involvement—whether detected surgically or radiologically—was associated with worse outcomes. Two-year overall survival differed markedly by para-aortic status (sPAOLN+ 40%, sPAOLN− 83%, iPAOLN+ 58%, iPAOLN− 69%; *p* < 0.001), and distant nodal metastases outside the pelvic and para-aortic fields were the dominant pattern of first recurrence among sPAOLN+ patients (4 of 11 recurrences, 36%). Although the group that underwent para-aortic lymphadenectomy as a whole showed better overall survival than the group staged only by imaging, this signal is difficult to interpret given non-random selection for surgery (frequent exclusion for comorbidities, extensive invasion, or positive para-aortic imaging) and substantial heterogeneity in treatment strategies over the long accrual period, including varying use of neoadjuvant chemotherapy, radical hysterectomy, and chemoradiation, which together limit the ability to attribute any survival differences to surgical staging. The principal criticism is that the survival comparison cannot be attributed to staging strategy, and so the main contribution of the study is in providing a contemporary estimate of the false-negative rate of imaging staging.

Chargari et al., 2016 [28]. Chargari and colleagues published a retrospective analysis of 186 women with LACC treated in a high-volume French center with concurrent chemoradiation and MRI-guided adaptive brachytherapy. The aim of the study was to evaluate the impact of primary retroperitoneal para-aortic lymphadenectomy (PAL) after PET-CT-negative para-aortic staging on disease-free survival outcomes. Patients were grouped into PET-CT negative with retroperitoneal lymphadenectomy (n = 119), PET-CT negative without surgical staging (n = 50), and PET-CT positive treated with extended-field chemoradiation (n = 17). Among PET-CT negative patients, PAL identified occult para-aortic metastases in 7.6% overall and in 15.8% of patients with pelvic nodal uptake, triggering extended-field irradiation. At 3 years, DFS (80% vs. 58%) and distant failure-free survival (87% vs. 67%) were significantly higher in the PAL than in the no-PAL group, while rates of local and locoregional failure were similar, suggesting that tailored field extension based on histologic para-aortic status may reduce distant, particularly para-aortic, relapses rather than improving pelvic control. Multivariable Cox models adjusting for pelvic nodal uptake and high-risk clinical target volume confirmed an association between PAL and better DFS and lower risk of distant failure (HR 0.47 for both endpoints). These findings suggest a possible benefit of tailored field extension guided by histologic para-aortic status; however, residual confounding and selection bias preclude a causal attribution. The single-center IGABT-specialized setting, baseline imbalances favoring the surgical arm (younger age, higher radiotherapy doses), and non-random allocation can overestimate the generalizable benefit of surgical staging.

Pomel et al., 2017 [29]. Pomel and colleagues compared two institutional strategies for para-aortic staging in 187 women with FIGO 2009 locally advanced cervical cancer treated with cisplatin-based chemoradiation and brachytherapy across two centers in France. In one center, para-aortic status was defined primarily by PET-CT (with extended fields for para-aortic or high pelvic nodal disease), whereas in the second center, all patients underwent laparoscopic para-aortic lymphadenectomy to guide radiotherapy fields. Despite similar FIGO stage distribution and comparable use of concurrent chemoradiation and extended-field RT, patients managed at the PET-CT center had significantly better outcomes, with 5-year DFS of 70.5% versus 49.2% and 5-year OS of 85.1% versus 63.8%, and multivariable models adjusting for World Health Organization (WHO) performance status, para-aortic involvement, brachytherapy use, completion surgery, stage, and histology still revealed a two-fold increased risk of recurrence and death among those treated in the surgical-staging center. However, the staging strategy was completely confounded by center, and important imbalances—including younger age, better WHO status, lower brachytherapy utilization and higher rates of completion surgery in the laparoscopic cohort—together with the absence of patient-level randomization, unreported time-to-treatment, and evolving techniques over the long accrual period, mean that the observed detriment with surgical staging cannot be directly attributed to para-aortic lymphadenectomy.

Dabi et al., 2018 [30]. Dabi and colleagues reported a multicenter French (FRANCOGYN) retrospective cohort of 647 women with FIGO 2009 IB2 or higher cervical cancer, all treated with concurrent chemoradiation and pre-treatment imaging (CT in 42.7%, PET-CT in 57.3%), and who were negative for para-aortic metastases. The aim was to compare oncologic outcomes between patients who underwent pre-treatment laparoscopic para-aortic lymphadenectomy (n = 377) and those staged by imaging alone (n = 270). Surgical staging identified occult para-aortic metastases in 47 of 377 (12.5%) patients, with 4.8% peri-operative and 13.3% post-operative complications of any severity. After a mean follow-up of 38.1 months, multivariable analysis using a Cox proportional hazards model identified surgical staging as an independent favorable factor for both disease-free survival (HR 0.64, 95% CI 0.46–0.89, *p* = 0.008) and overall survival (HR 0.43, 95% CI 0.27–0.68, *p* < 0.001), alongside intracavitary brachytherapy (HR 0.7, 95% CI 0.5–1.0 for DFS; HR 0.6, 95% CI 0.4–0.9 for OS). A para-aortic radiotherapy boost was administered in only 23.6% of patients overall, with no significant between-arm difference, and patterns of failure were broadly similar between groups, suggesting that the survival signal reflects a combination of more accurate risk stratification, a potential therapeutic effect of removing small-volume para-aortic disease, and center-level practice patterns rather than a protocol-driven change in radiation fields. Given the retrospective design, the center-driven nature of staging decisions, the mixed use of CT and PET-CT, and the absence of standardized extended-field RT indications, these results, suggesting possible survival in favor of surgical staging in selected patients, have to be interpreted as hypothesis-generating and should not be interpreted as a causal effect.

On a very important note, the Pomel [29], Chargari [28], and Dabi [30] studies share investigators and patient populations from a small group of French academic centers (Toulouse, Villejuif, and the FRANCOGYN network), which should be considered alongside when weighing the consistency of their findings.

González-Benítez et al., 2019 [31]. González-Benítez and colleagues published a single-center retrospective cohort of 74 patients with FIGO IIA2–IVA cervical cancer treated between 2005 and 2012. The primary aim was to define the influence of staging para-aortic lymphadenectomy on patient survival; this was done by comparing para-aortic staging by imaging (CT/MRI/PET-CT; n = 31) with laparoscopic lymphadenectomy (n = 43) before chemoradiation. Surgical staging detected para-aortic metastases in more cases (48.8% versus 33.3% by imaging), led to significantly more extended-field radiotherapy (44.2% vs. 19.4%) and higher radiation doses (50.5 vs. 45.5 Gy), and was associated with a 9.3% severe complication rate. Despite these differences, median progression-free survival (14.4 vs. 17.4 months) and overall survival did not differ significantly between groups. The authors concluded that para-aortic lymphadenectomy does not seem to yield a survival benefit, although it was associated with higher sensitivity for detecting occult para-aortic metastasis. Main limitations of this study include its small sample size, short follow-up, baseline imbalances favoring the imaging group (older age), higher chemotherapy use in the surgical arm, and absence of multivariable adjustment.

Yang et al., 2020 [32]. Yang and colleagues published a single-institution retrospective propensity score-matched analysis of 105 patients (35 surgical, 70 imaging) with FIGO 2009 IB2–IIIB cervical cancer treated with concurrent chemoradiation and brachytherapy. The aim was to compare oncologic outcomes between pre-treatment surgical (minimally invasive or open pelvic ± para-aortic lymphadenectomy) and imaging (CT, MRI, or PET-CT) lymph-node assessment. Surgical staging modified treatment in 31.4% (adding extended field radiation or radiotherapy boosts), revealed imaging sensitivity of 62.5% for para-aortic nodes, and was associated with a 14-day radiation treatment delay. After matching on stage, histology, and para-aortic status, no statistically significant difference in five-year PFS (62.6% vs. 72.4%) or OS (70.2% vs. 70.5%) was observed, as well as no differences in locoregional or distant recurrence patterns. Para-aortic metastasis emerged as the dominant adverse prognostic factor (HR 2.76 for PFS, 3.46 for OS). The principal criticisms are the small sample size and single-institution design; the take-away is that, despite better detection of occult disease, surgical staging did not improve survival in this matched contemporary cohort.

Nasioudis et al., 2022 [34]. Nasioudis and colleagues queried the U.S. National Cancer Data Base (NCDB) for women with FIGO 2009 IB2–IVA cervical cancer receiving definitive chemoradiation between 2010 and 2015 (n = 3540). The aim of the study was to compare overall survival between para-aortic surgical staging (n = 333; 9.4%) and imaging-based staging. Surgical staging detected higher para-aortic positivity (27.3% vs. 13.2%) but was associated with a longer diagnosis-to-radiotherapy interval (55 vs. 38 days; difference of 17 days). Despite these differences and patient imbalances (younger, healthier surgical cohort), 4-year OS was similar between groups (62.9% vs. 63.0%, *p* = 0.80, without a prespecified equivalence margin), with no surgical benefit on multivariable analysis (HR 1.07, 95% CI 0.88–1.31) or in subgroups by stage or facility. Sensitivity analyses restricted to patients receiving brachytherapy or to those with ≥10 lymph nodes removed confirmed the null finding. The principal criticisms include the inherent limitations of administrative datasets (no central pathology, no PET-CT data, limited information on radiotherapy technique). However, the very low utilization of surgical staging in U.S. routine practice (9.4%) and the consistent null effect underscore that, in this real-world population, surgical staging has neither been widely adopted nor been associated with a survival advantage.

Jiang et al., 2024 [33]. Jiang and colleagues reported the most recent comparative study in this field: a single-center Chinese retrospective cohort of 171 patients with imaging-confirmed pelvic-node-positive FIGO 2009 IB2/IIA2–IVA LACC, comparing laparoscopic combined para-aortic and pelvic lymphadenectomy prior to concurrent chemoradiation (n = 58) with CT-based imaging staging (n = 113). The aim of the study was to compare survival and treatment-related outcomes between strategies in patients with imaging-positive pelvic nodes. Surgical staging identified occult para-aortic metastases in 34.5% of patients and re-classified pelvic nodal status in 12.2% of cases that had been called positive on imaging; the procedure was feasible (median operative time 120 min, 50 mL blood loss) and morbidity was low, with lymphatic cysts in 9.3%. Initiation of radiotherapy was delayed by a median of 10.2 days in the surgical arm without translating into excess grade 3–4 toxicity. After 52 months of median follow-up, surgical staging was not associated with improved five-year PFS (70.7% vs. 64.6%, HR 0.80, 95% CI 0.45–1.41, *p* = 0.43) or OS (75.9% vs. 69.7%, HR 0.77, 95% CI 0.41–1.43, *p* = 0.40). Major limitations include the single-center retrospective design and the use of CT rather than PET-CT for imaging staging—both of which likely overestimate the diagnostic gap that surgical staging can close. Nonetheless, the study aligns with the cumulative evidence of this review: surgical staging consistently detects more occult disease without translating into a survival benefit in patients with pelvic-node-positive LACC.

## 4. Discussion

Across two randomized controlled trials, nine retrospective cohorts, and one population-based analysis, pre-treatment surgical para-aortic staging in LACC consistently increases detection of occult para-aortic disease (upstaging up to 48% of patients) and prompts more frequent use of extended-field radiotherapy (18–44%), but does *not seem* to translate into a reproducible advantage in progression-free or overall survival outcomes when compared with imaging-based staging. The two randomized trials illustrate the opposite ends of the treatment-era spectrum: Lai 2003 [24] demonstrated harm in the surgical arm under outdated radiotherapy and chemotherapy conditions, whereas UTERUS-11—the only modern phase III trial—found no significant difference in the primary endpoint of progression-free survival, with exploratory post hoc signals (cancer-specific survival and the stage-IIB subgroup) that warrant hypothesis-testing in future trials but should not be interpreted as confirmatory. A previously published systematic review included a meta-analysis of four retrospective cohorts and one randomized controlled trial comparing surgical versus imaging-based para-aortic staging [38]. Notably, that pooled analysis combined heterogeneous designs (one randomized trial and four retrospective cohorts), and the wide confidence intervals (PFS HR 1.13, 95% CI 0.73–1.74; OS HR 1.06, 95% CI 0.66–1.69) and high statistical heterogeneity (I^2^ = 75% for both endpoints) indicate substantial imprecision rather than confirmatory equivalence. The hazard ratios are reported with imaging-based staging as the reference; values above 1 would indicate worse outcomes with surgical staging, but the wide confidence intervals encompass both directions. This evidence is therefore best interpreted as low-certainty and heterogeneous. In contrast, we consider that the substantial clinical and methodological heterogeneity across these studies, as well as the mixing of randomized and observational data, limits the validity of pooled effect estimates and argues against quantitative synthesis.

In terms of the procedures, surgical staging was feasible and safe in contemporary minimally invasive series, with intraoperative complication rates of 1.6–6.9% and severe complications (Clavien-Dindo grade ≥ III) of 8.6–9.3%, in contrast to historical transperitoneal series, which reported markedly higher rates of late radiation-related bowel complications (up to 36.8% in Chen 2012 [26]). Surgical staging delayed initiation of definitive radiotherapy by a reported median of 10–22 days in modern cohorts; the survival impact of these delays remains uncertain and has not been formally analyzed as a time-dependent exposure in the available comparative studies. Importantly, across several series, pathologic para-aortic node involvement emerged as a dominant adverse prognostic factor with hazard ratios for recurrence and death in the range of 2–3.5 [32]. The available data suggest that para-aortic involvement is a strong adverse prognostic factor; whether better systemic therapy, altered radiation fields, surgery, or combinations improve outcomes remains uncertain. In this context, modern IMRT-based extended-field chemoradiation and risk-adapted elective para-aortic irradiation, guided by PET-CT and EMBRACE-II/ESGO–ESTRO criteria [10,14], may potentially mitigate some of the historical trade-off between undertreatment and overtreatment without the added morbidity and possible delays associated with systematic para-aortic lymphadenectomy.

### 4.1. An Evolving Systemic-Therapy Landscape

A consideration that has not been addressed in the presented studies is that the contemporary systemic-therapy landscape in LACC has changed substantially. The INTERLACE trial demonstrated that induction chemotherapy with carboplatin and paclitaxel followed by chemoradiation improves progression-free and overall survival compared with chemoradiation alone [39], and the KEYNOTE-A18 trial demonstrated that the addition of pembrolizumab to chemoradiation improves both progression-free and overall survival in high-risk LACC, including in patients with positive lymph nodes [9]. Both regimens deliver systemic therapy that is expected to address occult micro-metastatic disease irrespective of whether it was detected at staging. In this context, the diagnostic gain of surgical staging is partly absorbed by upfront systemic intensification, and the incremental therapeutic value of detecting and irradiating occult para-aortic disease is apparently smaller than it would have been in the pre-INTERLACE/pre-KEYNOTE-A18 era. This evolving therapeutic backdrop is a further argument in favor of restricting routine surgical staging to prospective trials designed to assess its incremental value in the context of modern systemic therapy.

At the heart of the contemporary clinical decision lies a defined trade-off: prophylactic elective para-aortic extended-field radiotherapy based on EMBRACE-II nodal-burden criteria avoids surgical morbidity at the cost of irradiating an unselected proportion of patients without occult disease, whereas pre-treatment surgical staging selects patients for tailored EFRT at the cost of operative morbidity, delays and the resource demands of minimally invasive surgical expertise.

### 4.2. Limitations of the Available Evidence

From a target-trial perspective, the ideal comparison would be a randomized assignment, at the time of diagnosis (time zero), to either pre-treatment minimally invasive para-aortic surgical staging or imaging-based staging (preferably PET-CT), followed by EMBRACE-II-standard chemoradiation with image-guided brachytherapy and pre-specified risk-adapted radiation fields, with progression-free and overall survival as primary endpoints assessed with adequate follow-up and explicit handling of censoring. None of the included studies fully approximates this target: Lai used outdated radiation and systemic therapy with delayed RT in the surgical arm; UTERUS-11 randomized appropriately but lacked routine PET-CT and was underpowered; the observational cohorts assign staging by center, clinician preference, or registry coding, with heterogeneous radiation-field decisions, follow-up duration, and outcome definitions; and the NCDB analysis cannot adjudicate radiation technique or recurrence. Each study therefore deviates from the target comparison in identifiable ways, which we have flagged paragraph-by-paragraph above and summarize descriptively in Table 2, emphasizing that this table is an author-developed descriptive matrix to aid critical appraisal and is not a formal risk-of-bias assessment, such as RoB 2 or ROBINS-I.

Study design and patient selection, variable radiotherapy and chemotherapy regimens, and surgical staging approach are the most relevant factors. Some studies pre-date contemporary standards: outdated chemotherapy (5-fluorouracil, hydroxyurea), four-field radiotherapy without brachytherapy, and open surgical approaches are common in Lai, Gold, Chen, Vandeperre, Pomel, Dabi, and Yang. Several studies used adjuvant chemotherapy after chemoradiation [19,20,22,35]. Unfortunately, this approach is not supported by the OUTBACK trial, a prospective randomized clinical trial evaluating adjuvant chemotherapy after concurrent chemo-radiotherapy in patients with LACC, which showed no survival benefit and increased toxicity [40]. To date, this intervention is not a standard practice. Inherently, retrospective cohorts are affected by selection bias and heterogeneous eligibility, with patients with comorbidity or imaging-positive para-aortic disease often systematically excluded from the surgical arm as seen in some studies [19,21,22,23,27,36]. Several studies included patients with imaging-positive para-aortic nodes in the surgical arm or combined surgical staging with pelvic debulking, completion hysterectomy, or prophylactic extended-field radiotherapy without strong supporting evidence [19,21]. Center-level practice patterns appear to confound apparent advantages or disadvantages of lymphadenectomy (Pomel, Chargari, Dabi—three studies that, as noted above, share investigators and national academic networks in France). Finally, only one trial (UTERUS-11) used a randomized design in the modern era, and it remained underpowered due to slow accrual and limited PET-CT availability.

### 4.3. Ongoing Trials

Two ongoing phase III clinical trials are designed to test the clinical question of whether surgical staging provides incremental value in selected patients in the contemporary treatment era.

The PAROLA trial (NCT05581121) [17] is randomizing 510 patients with FIGO 2018 stage IIIC1r cervical cancer (PET-CT-positive pelvic nodes, PET-CT-negative para-aortic nodes) to minimally invasive surgical para-aortic staging with tailored chemoradiation versus PET-CT-based staging with prophylactic extended-field radiotherapy if there are three or more positive pelvic nodes. It was developed hypothesizing a 3-year DFS superiority (70% vs. 60%, HR 0.70) via occult detection (estimating a 20 to 30% upstaging rate) without overtreatment toxicity. PAROLA mandates contemporary EMBRACE-II/ESGO–ESTRO standards (IMRT, image-guided brachytherapy, and cisplatin) and includes ancillary studies of sentinel nodes, radiomics, circulating tumor DNA, as well as quality-of-life and cost outcomes. This study has been recruiting participants since December 2023, with full accrual expected by 2027 and final results by 2033, as noted in ClinicalTrials.gov (https://clinicaltrials.gov/study/NCT05581121 (accessed on 10 June 2026)).

The CQGOG0103 trial (NCT04555226) [22] addresses a complementary population—FIGO IIICr patients with bulky imaging-positive nodes (short diameter ≥ 15 mm)—randomizing 452 patients to chemoradiation versus pelvic and para-aortic lymphadenectomy followed by chemoradiation. Primary and secondary outcomes include 2-year progression-free and 3 year-overall survival. It is currently recruiting and study completion is estimated by 2032 (https://clinicaltrials.gov/study/NCT04555226 (accessed on 10 June 2026)). PAROLA and CQGOG0103 [21,22] are designed to test whether surgical staging can add incremental value to modern chemoradiation in terms of oncological outcomes in similar but not equivalent populations; nonetheless, their results should clarify whether surgical staging adds value in contemporary treatment pathways and may inform future guideline updates.

## 5. Conclusions

In LACC, pre-treatment surgical para-aortic staging improves anatomic and prognostic information but has not shown a consistent survival advantage over imaging-based staging combined with contemporary chemoradiation. Current comparative evidence does not support routine surgical staging, so until results from ongoing phase III trials like PAROLA or CQGOG0103 are available, this method should be considered an individualized option within multidisciplinary decision-making at experienced centers. For resource-constrained settings where PET-CT, image-guided adaptive brachytherapy and minimally invasive surgical expertise are limited, the available evidence supports MRI- or contrast-enhanced CT-based staging combined with risk-adapted elective para-aortic extended-field radiotherapy according to EMBRACE-II nodal-burden criteria for definitive treatment.

## Figures and Tables

**Table 2:** Descriptive table of methodological limitations across included studies, grouped by domain *.

		A. Design and Patient Selection			B. Radiation and Systemic Therapy					C. Surgical/Staging Approach	
Study	Design	A1	A2	A3	B1	B2	B3	B4	B5	C1	C2
**Lai et al., 2003** [24]	RCT	**○**	**○**	**—**	**●**	**●**	**●**	**●**	**○**	**●**	**○**
**Marnitz et al., 2020 (UTERUS-11)** [6]	RCT	**●**	**●**	**—**	**○**	**○**	**○**	**○**	**○**	**○**	**○**
**Gold et al., 2008** [25]	Pooled GOG post hoc	**●**	**○**	**●**	**●**	**○**	**●**	**●**	**○**	**●**	**○**
**Chen et al., 2012** [26]	Retrospective	**○**	**●**	**●**	**?**	**●**	**?**	**○**	**○**	**○**	**○**
**Vandeperre et al., 2015** [27]	Retrospective	**○**	**●**	**●**	**?**	**○**	**?**	**?**	**○**	**○**	**●**
**Chargari et al., 2016** [28]	Retrospective	**○**	**○**	**●**	**○**	**○**	**○**	**○**	**○**	**○**	**○**
**Pomel et al., 2017** [29]	Retrospective	**○**	**●**	**●**	**○**	**○**	**○**	**●**	**○**	**○**	**●**
**Dabi et al., 2018** [30] **(FRANCOGYN)**	Retrospective	**○**	**○**	**●**	**○**	**○**	**●**	**●**	**○**	**○**	**●**
**González-Benítez et al., 2019** [31]	Retrospective	**○**	**●**	**●**	**○**	**○**	**○**	**●**	**●**	**○**	**○**
**Yang et al., 2020** [32]	Retrospective (PSM)	**○**	**●**	**○**	**○**	**○**	**●**	**●**	**○**	**●**	**○**
**Jiang et al., 2024** [33]	Retrospective	**○**	**●**	**●**	**○**	**○**	**?**	**○**	**○**	**○**	**○**
**Nasioudis et al., 2022** [34]	Population-based (NCDB)	**?**	**?**	**●**	**?**	**?**	**?**	**?**	**○**	**?**	**?**

**Legend: ●** = limitation present (documented in the original publication); **○** = limitation absent (explicitly addressed or avoided in the original study); **?** = not clearly reported or unclear from the original publication; **—** = not applicable to this study design. **Domain item definitions: A. Design and patient selection: A1—**No pre-operative imaging in surgical arm. **A2—**Imaging-positive para-aortic LN allowed in surgical arm. **A3—**Sicker/advanced-stage patients excluded from surgical arm (selection bias). **B. Radiation and systemic therapy: B1—**Four-field (non-IMRT) external-beam radiotherapy. **B2—**Radiotherapy without concurrent chemotherapy in >10%. **B3—**External-beam RT without brachytherapy in >10%. **B4—**Adjuvant chemotherapy after CCRT in >10%. **B5—**Disproportionate (>50%) prophylactic para-aortic RT in imaging arm. **C. Surgical/staging approach: C1—**Open (non-MIS) surgical staging in >10%. **C2—**Hysterectomy as part of primary management. * This table is an author-developed descriptive matrix to aid critical appraisal and is not a formal risk-of-bias assessment such as RoB 2 or ROBINS-I.

## Data Availability

No new data were generated for this narrative review. All studies discussed are publicly available through the cited journals.

## Abbreviations

The following abbreviations are used in this manuscript:

**18F****-FDG**	18-Fluorodeoxyglucose
**5-FU**	5-Fluorouracil
**CCRT**	Concurrent chemoradiotherapy
**CI**	Confidence interval
**CQGOG0103**	Chongqing Gynecologic Oncology Group 0103 trial
**CRT**	Chemoradiotherapy
**CSS**	Cancer-specific survival
**CT**	Computed tomography
**DFS**	Disease-free survival
**EBRT**	External-beam radiotherapy
**EFRT**	Extended-field radiotherapy
**EMBRACE**	Image-guided intensity-modulated External Beam radiochemotherapy and MRI-based adaptive Brachytherapy in locally Advanced CErvical cancer
**ENGOT**	European Network of Gynaecological Oncological Trial Groups
**ESGO**	European Society of Gynaecological Oncology
**ESP**	European Society of Pathology
**ESTRO**	European Society for Radiotherapy and Oncology
**EXP**	Extraperitoneal (approach)
**FFS**	Failure-free survival
**FIGO**	International Federation of Gynecology and Obstetrics
**FRANCOGYN**	French research network in gynaecological oncology
**GCIG**	Gynecologic Cancer InterGroup
**GINECO**	Groupe d’Investigateurs Nationaux pour les Études des Cancers Ovariens
**GOG**	Gynecologic Oncology Group
**Gy**	Gray (radiation dose unit)
**HDI**	Human Development Index
**HDR**	High-dose rate (brachytherapy)
**HR**	Hazard ratio
**ICI**	Immune checkpoint inhibitor
**IGABT**	Image-guided adaptive brachytherapy
**IMA**	Inferior mesenteric artery
**IMRT**	Intensity-modulated radiotherapy
**INTERLACE**	Induction chemotherapy plus chemoradiation in locally advanced cervical cancer trial
**iPAOLN**	Imaging para-aortic lymph node
**KEYNOTE-A18**	KEYNOTE-A18/ENGOT-cx11/GOG-3047 trial
**LACC**	Locally advanced cervical cancer
**LAP**	Laparoscopic/laparoscopy
**LN**	Lymph node
**MRI**	Magnetic resonance imaging
**NACT**	Neoadjuvant chemotherapy
**NCCN**	National Comprehensive Cancer Network
**NCDB**	National Cancer Data Base
**OR**	Odds ratio
**OS**	Overall survival
**PAL**	Para-aortic lymphadenectomy
**PALN**	Para-aortic lymph node
**PALND**	Para-aortic lymph node dissection
**PAROLA**	PARa-aOrtic LymphAdenectomy in Locally Advanced Cervical Cancer trial
**PET-CT**	Positron emission tomography–computed tomography
**PFS**	Progression-free survival
**PS**	Performance status
**PSM**	Propensity score-matched
**RCT**	Randomized controlled trial
**RT**	Radiotherapy
**SEGO**	Spanish Society of Gynaecology and Obstetrics
**sPAOLN**	Surgical para-aortic lymph node
**TLSPAD**	Transperitoneal laparoscopic para-aortic dissection
**UTERUS-11**	UTERUS-11 randomized trial (Marnitz et al., 2020 [6])
**WHO**	World Health Organization
